# Relationships between humans and ungulate prey shape Amur tiger occurrence in a core protected area along the Sino‐Russian border

**DOI:** 10.1002/ece3.4620

**Published:** 2018-10-30

**Authors:** Wenhong Xiao, Mark Hebblewhite, Hugh Robinson, Limin Feng, Bo Zhou, Pu Mou, Tianming Wang, Jianping Ge

**Affiliations:** ^1^ State Key Laboratory of Earth Surface Processes and Resource Ecology, Ministry of Education Key Laboratory for Biodiversity Science and Engineering, College of Life Sciences Beijing Normal University Beijing China; ^2^ Wildlife Biology Program, Department of Ecosystem and Conservation Sciences, W.A. Franke College of Forestry and Conservation University of Montana Missoula Montana; ^3^ Institute of Zoology Chinese Academy of Sciences Beijing China; ^4^ Panthera New York New York

**Keywords:** abundance, camera traps, large carnivore, occupancy model, predator–prey, tiger

## Abstract

Large carnivore populations are globally threatened by human impacts. Better protection could benefit carnivores, co‐occurring species, and the ecosystems they inhabit. The relationship between carnivores and humans, however, is not always consistent in areas of high human activities and is often mediated through the effects of humans on their ungulate prey. To test assumptions regarding how prey abundance and humans affect carnivore occurrence, density, and daily activity patterns, we assessed tiger–prey–human spatiotemporal patterns based on camera‐trapping data in Hunchun Nature Reserve, a promising core area for tiger restoration in China. Our study area contained seasonally varying levels of human disturbance in summer and winter. We used N‐mixture models to predict the relative abundance of ungulate prey considering human and environmental covariates. We estimated tiger spatial distribution using occupancy models and models of prey relative abundance from N‐mixture models. Finally, we estimated temporal activity patterns of tigers and prey using kernel density estimates to test for temporal avoidance between tigers, prey, and humans. Our results show that human‐related activities depressed the relative abundance of prey at different scales and in different ways, but across species, the relative abundance of prey directly increased tiger occupancy. Tiger occupancy was strongly positively associated with the relative abundance of sika deer in summer and winter. The crepuscular and nocturnal tigers also apparently synchronized their activity with that of wild boar and roe deer. However, tigers temporally avoided human activity without direct spatial avoidance. Our study supports the effects of humans on tigers through human impacts on prey populations. Conservation efforts may not only target human disturbance on predators, but also on prey to alleviate human–carnivore conflict.

## 1 INTRODUCTION

Wide‐ranging large carnivores are commonly recognized as umbrella species, as they usually require large areas of habitat due to high metabolic demands and sensitivity to human activity, and their conservation is thus thought to provide benefits for other species (Ripple et al., 2014). However, with human population increases and urbanization over the past century, large carnivore populations and their habitats have declined globally (Ripple et al., 2014). Carnivore–human coexistence is becoming an increasingly important issue in wildlife and ecosystem conservation (Graham, Beckerman, & Thirgood, 2005; Oriol‐Cotterill, Valeix, Frank, Riginos, & Macdonald, 2015). Effective conservation of large carnivores could facilitate protection of the landscapes they inhabit as well as co‐occurring species (Thornton et al., 2016).

Human‐induced habitat degradation and loss, prey depletion, and poaching are widely recognized as the main threats to large carnivores (Karanth, Chundawat, Nichols, & Kumar, 2004; Wolf & Ripple, 2016). Humans cause large carnivore mortality through poaching and accidental snaring (Kerley et al., 2002; Lindsey et al., 2013), and livestock predation that leads to retaliatory killing or problem carnivore removal (Holmern, Nyahongo, & Røskaft, 2007). Humans also affect large carnivores through direct poaching of their ungulate prey (Datta, Anand, & Naniwadekar, 2008). As an indirect result of carnivore–human conflict, carnivores often avoid human activity across spatiotemporal scales to reduce the risk of conflict with humans (Carter, Shrestha, Karki, Pradhan, & Liu, 2012; Chanchani, Noon, Bailey, & Warrier, 2016; Hebblewhite et al., 2014). Whether large carnivores can avoid human activity enough to reduce their direct mortality from humans is a key conservation question and often hotly debated. For example, recent analyses in Nepal suggested the potential for tiger (*Panthera tigris*) human coexistence (Carter et al., 2012), but their conclusions were widely criticized (Karanth et al., 2013), and one of the reasons was their failure to explicitly consider prey abundance.

Prey abundance is perhaps the most important nonhuman factor affecting large carnivore occurrence (Karanth & Stith, 1999), density (Karanth et al., 2011), habitat selection, energetics, and reproduction (Miller et al., 2013). Large herbivores are the major food resource for large carnivores and are themselves also vulnerable to human‐induced disturbance (Proffitt, Gude, Hamlin, & Messer, 2013). A major impact on large carnivores is the indirect effects of humans via depletion of their large ungulate prey (Ripple et al., 2015). Large herbivores have also been shown to modify both spatial and temporal activity patterns to avoid human activity and predation risk (Podgórski et al., 2013). Temporally, predators tend to have similar activity patterns as their primary prey species and lower overlap with less frequently consumed prey species (Ramesh, Kalle, Sankar, & Qureshi, 2012). As a major source of human–tiger conflict, livestock grazing can also directly influence large carnivores (Karanth et al., 2011) as well as compete with wild herbivores for forage resulting in potential declines in large herbivores (Ripple et al., 2015). Therefore, livestock activity can also impact large carnivores and their prey (Berger, Buuveibaatar, & Mishra, 2013; Fleischner, 1994).

**Figure 1:**
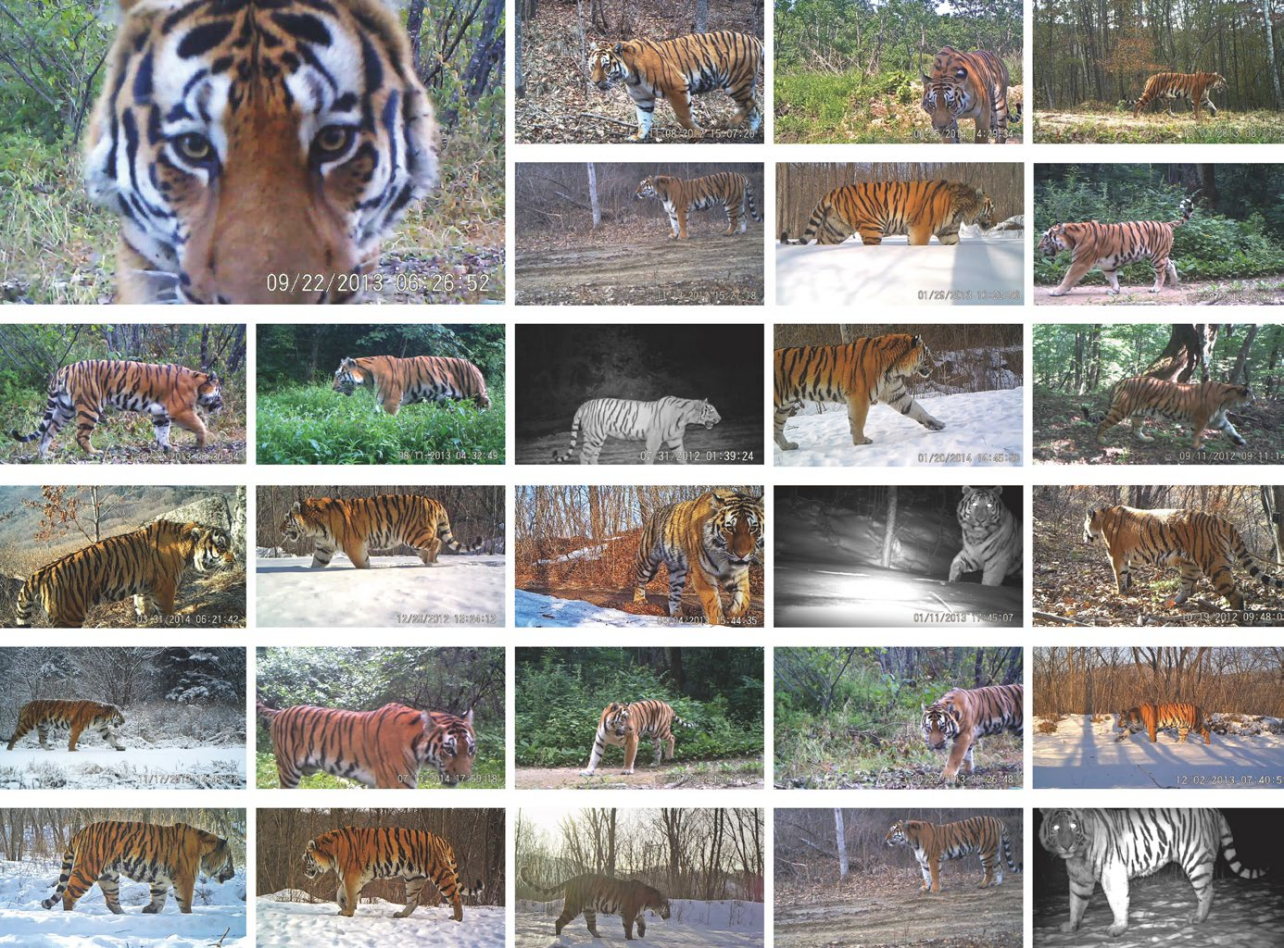
Amur tigers recorded by the camera trap we set up in Hunchun area

We used Amur tiger (*Panthera tigris altaica*) as a model to investigate how large carnivores interact with prey and human disturbance at different spatiotemporal scales. Northeast China was the Amur tiger's primary habitat 100+ years ago. Today however, due to increasing human‐related activities and prey reduction, the majority of the Amur tiger population occurs in the Russia Far East. In the last decade, through dispersal from Russia (Wang et al., 2016) as well as recovering potential habitat in China (Hebblewhite et al., 2012), a small but growing population of approximately 12–16 individuals now exists along the Russian border in the Hunchun Nature Reserve which is the core habitat for recovery tigers in China (Figure 1; Wang et al., 2018; Xiao et al., 2016). The Chinese government has prioritized tiger population recovery through science‐based conservation strategies including a logging ban in tiger habitat, and the establishment of the first national park focused on tiger and leopard (*Panthera pardus*) conservation (McLaughlin, 2016). Indeed, through the Global Tiger Initiative, the Chinese government has formally committed, along with 12 other tiger range countries, in doubling wild tiger numbers by 2022. However, with higher levels of human‐related disturbance (e.g., human and cattle activities) especially in summer, and lower wild prey density compared with Russia (Soh et al., 2014), how tigers and wild prey will respond to human activities in their core protected areas in China (Wang et al., 2018) is an important knowledge gap that would help optimize conservation investments and facilitate restoration planning.

We tested the overall working hypothesis that tiger occurrence and abundance are driven by abundance of their primary prey and that human activities affected primarily prey abundance. We examined tiger–prey–human relationships at multiple spatial scales, as well as seasonally between summer and winter season to take advantage of reduced human activity during winter. Spatially, we tested how human disturbance influenced prey abundance and tiger occupancy and how this related to previously published estimates of spatial density in their core protected area habitat (Wang et al., 2018; Xiao et al., 2016). We also examined temporal partitioning by testing for daily activity overlap between tiger, wild prey, human, and cattle. We note we cannot test for direct effects of humans on tigers, which requires demographic data, but here focus on understanding indirect effects of human activity on tigers and their prey. We first estimated the relative effect of human‐related and environmental factors on the relative abundance of prey species by applying N‐mixture models to the main prey species of tigers in our study area (Kery, 2018; Royle, 2004). Previous research in China did not specifically address prey detection, or factors affecting prey abundance (Wang et al., 2018). We then used these relative abundance estimates as well as human and cattle detection frequencies as covariates in spatial occupancy models for tigers (MacKenzie et al., 2002). To test for temporal overlap between tigers, prey, and livestock, we applied kernel density estimation to estimate the daily temporal pattern of activity for tigers, three ungulate species as well as human and cattle activities in forest in order to measure the overlap (Linkie & Ridout, 2011; Ridout & Linkie, 2009) and interaction. Finally, because occupancy is not necessarily linearly related to density (Kéry & Royle, 2015; Steenweg, Hebblewhite, Whittington, Mckelvey, & Lukacs, 2018), we tested the relationship between tiger occupancy and a previously published spatial model of density developed from spatially explicit capture–recapture (SCR) model within Hunchun Nature Reserve (Xiao et al., 2016).

## 2 MATERIALS AND METHODS

### 2.1 Study area and camera trap design

**Figure 2:**
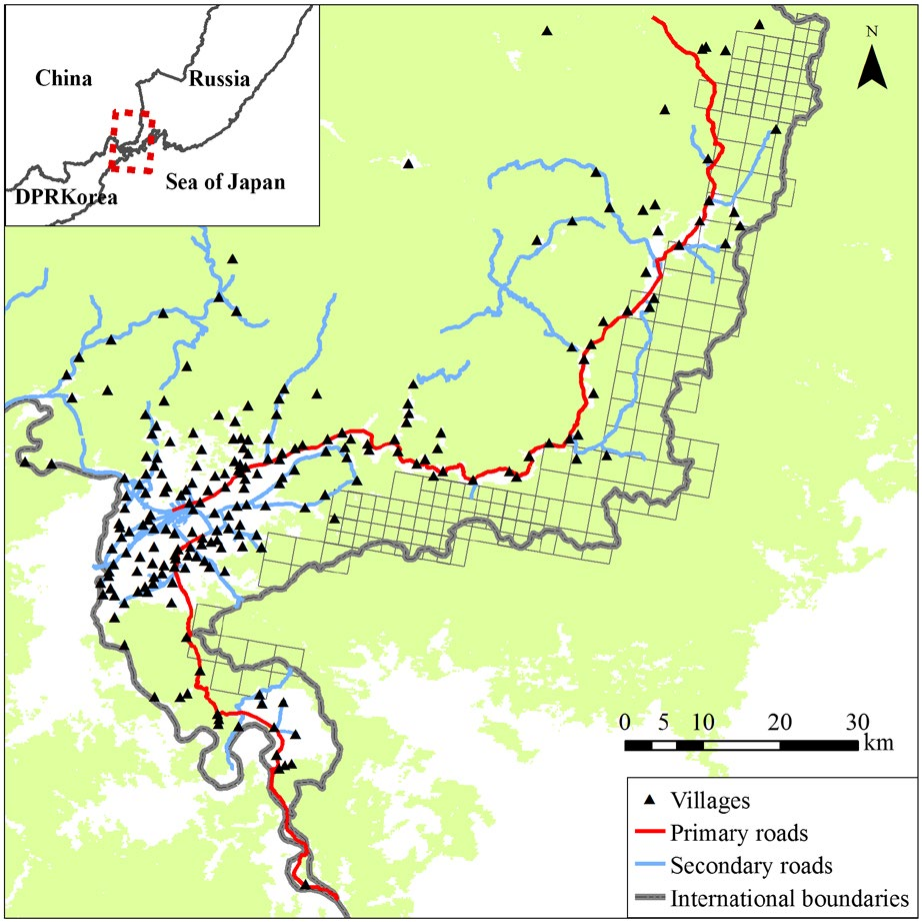
Study area location in Hunchun Nature Reserve in Northeast China and on the border of the Russian Far East, the Democratic People's Republic of Korea (DPR Korea). Our two scales of remote camera trap sampling design are also shown. The large grids are 3.6 × 3.6 km grids, and the small grids are 1.8 × 1.8 km subgrids. Inset shows the location of study area. Green area shows forest coverage

Our study area focused on the core habitat for recovering Amur tigers in China in the Hunchun Nature Reserve (HNR) located in eastern Jilin province, China (Figure 2, Wang et al., 2018). HNR is 1,087 km^2^, with an additional adjacent 418 km^2^ west of the region designated as community‐based natural resource management zone (Li, Zhang, Zhang, & Liu, 2008). With tiger recovery ongoing, our study certainly occurred during a dynamic, nonequilibrial period of predator–prey spatial and temporal dynamics. The study area is comprised of elevations from 5 to 973 m, a mix of forest types, and, despite being a nature reserve, contains significant human infrastructure and natural resource extraction. More than 14,000 people live in 29 villages within the reserve, and the average people density is 12 people/km^2^ (Han, Tong, Zhen, & Li, 2003). There are three kinds of roads in the study area (Figure 2), from paved highways to forest roads/trails to facilitate timber extraction. Cattles are grazed seasonally, and other nontimber forest products are harvested seasonally as well (e.g., Ginseng; see Xiao, 2011 for more details).

From December 2012 to July 2014, we divided potential tiger habitat in HNR into 3.6 × 3.6 km grids based on the home range of ungulate prey, including sika deer (*Cervus nippon*), wild boar (*Sus scrofa*), and roe deer (*Capreolus pygargus*), and set one remote camera in each grid cell (Figure 2). For ungulate monitoring at multiscales, we also created a finer 1.8 × 1.8 km subgrid in selected areas of known tiger occurrence based on previous studies (Xiao, 2014), placing an additional 2–3 cameras in each 3.6 × 3.6 km grid (Figure 2). Thus, we deployed cameras at a total of 163 locations representing 90 3.6 × 3.6‐km grids in winter and 166 locations representing 91 grids in summer. All cameras we used were the Ltl Acorn model manufactured by Ltl Acorn Electronics Co., Ltd., China. All cameras used a passive infrared (PIR) sensor set with a 0.8 s trigger speed. We deployed cameras along forest roads (*n* = 86 sites) and game trails (*n* = 80 sites) commonly used by tigers and ungulate across various biotic, abiotic, and anthropogenic conditions. Cameras were tied against tree trunks at a height of 0.4–0.8 m and were set to take 15‐s videos when triggered by a differential in heat and motion (e.g., PIR) between a subject and the background temperature. The delay between two consecutive triggers was set to 1 min. We maintained camera stations each 1–2 months. We classified an independent observation following O'Brien, Kinnaird, and Wibisono (2003) to avoid pseudoreplication (see below in N‐mixture modeling for details). We then divided data into winter (December 2012–April 2013 and November 2013–April 2014) and summer (May–October 2013 and May–July 2014) seasons based on timing of snow and associated human‐related activities.

### 2.2 Modeling relative prey abundance

We first developed relative abundance models of the main ungulate prey species for use in tiger occupancy models. Here, we focused on the three most important ungulate prey species for Amur tigers in Southwest Primorye Krai in Russia and China, sika deer, wild boar, and roe deer (Hebblewhite, Miguelle, Murzin, Aramilev, & Pikunov, 2011; Kerley et al., 2015; Xiao et al., 2014). We developed N‐mixture models at the 3.6 × 3.6 km scale. When we had subsampled at a higher spatial resolution within the intensive, 1.8 × 1.8 km sampling areas (Figure 2), we used all 2–4 available camera's in the 3.6 × 3.6 km N‐mixture model as spatial replica. We chose this approach instead of discarding data or developing multiscale models. We estimated the relative abundance of the three ungulate species’ using single‐season, single‐species N‐mixture models (Kéry & Royle, 2015) as a function of environmental covariates. For each species, we separated seasonal models for winter and summer.

N‐mixture models assume that all within‐site variation in counts is attributable to detection probability and no false positives occur (i.e., detecting individuals more than once or erroneously add some other species in the count) (Kéry & Royle, 2015). It was impossible to distinguish individuals of the three species of ungulates based on camera data. Therefore, we used N‐mixture models to estimate the relative abundance of prey as a surrogate for abundance (Kéry & Royle, 2015). In this context, recent simulations and statistical models confirm that N‐mixture models can provide reliable estimates of relative abundance despite the challenge of ensuring complete population closure within a sampling occasion (Arnold, 2010; Denes, Silveira, & Beissinger, 2015). For example, Barker, Schofield, Link, and Sauer (2018) showed that if detection probability is effectively modeled with covariates, count data (from sampling such as camera trapping) can provide a reliable index of relative abundance, regardless of assumptions about closure, especially with Poisson or zero‐inflated Poisson distribution for abundance (Joseph, Elkin, Martin, & Possingham, 2009; Kery, 2018). Applied N‐mixture models using camera data have been shown to be an effective method for ungulate population estimation (Keever et al., 2017).

For each camera site, we used a 10‐day period as the temporal sampling unit (i.e., survey occasion) and counted the maximum number of individuals visible simultaneously in each “event,” an independent 15‐s video (we considered “events” occurring >30 min apart as independent), and then calculated the accumulated individuals within each 10‐day occasion. We standardized all quantitative covariates to improve estimation and then conducted all analysis in the R package “unmarked” (Fiske & Chandler, 2011).

#### 2.2.1 Ungulate detection probability

We first obtained the best detection probability model for each season and each species while including no covariates on relative abundance part of the N‐mixture model following Doherty, White, and Burnham (2012) and Kéry and Royle (2015). The covariates for site *i* and survey occasion *j* used to model detectability included camera days (total days each camera was in operation) as a measure of effort, and forest road or trail (trails hereafter) width, including a quadratic of trail width (Table 1). The mean trail width of the camera trap set along forest roads was 2.6 m (ranges 1.6–6 m), while the mean trail width of the camera trap set along game trails was 0.6 m (ranges 0–1.8 m). We suspected that wider trails might have a higher probability of detection as animals usually take longer to cross the detection zone of the camera, but if the trail was too wide for the camera may not be triggered, hence we also tested a quadratic effect. In addition, trail width effectively represented trail type (i.e., 0.6 m vs. 2.6 m for trails vs. roads, see above). We also allowed for time‐varying detection probabilities within different occasions following the general advice of Doherty et al. (2012). As the relationship between the occasion and detection probability might be linear or variable among different occasions, we treated it as a continual covariate and a categorical covariate with 10‐, 30‐, or 60‐day intervals (Table 1). We tested for correlation between all covariates using a Pearson correlation and dropped covariates that were correlated with |r|> 0.6. We then selected the best occasion‐specific detection probability models using Akaike information criterion (AIC) to obtain corresponding time period covariates (Burnham & Anderson, 2002). We then selected the top detection probability model from all possible combinations of covariates.

**Table 1:** Covariates category, names, and the data sources for ungulate N‐mixture models

Covariate category/name	Sources
Relative abundance covariates	
Biotic covariates	
Forest type[Link], classified as broadleaf deciduous forest, mixed Korean pine‐deciduous forest, and mixed Korean pine‐spruce forest	Moderate‐resolution Imaging Spectroradiometer (MODIS) in 2009
Abiotic covariates	
Elevation[Link]	30 m DEM, SRTM
Slope[Link] (degree)	30 m DEM, SRTM
Northness[Link], range from −1 (aspect toward south) to 1 (aspect toward north)	30 m DEM, SRTM
Distance to frontier (km)	Forest inventory data in Changbaishan Mountain
Distance to river (km)	Forest inventory data in Changbaishan Mountain
Anthropogenic covariates	
Human activity	Count of independent observations of humans at each camera site
Cattle activity (summer season only)	Count of independent observations of cattle at each camera site
Road density[Link] (km/sq kilometer)	Forest inventory data in Changbaishan Mountain
Human population density[Link] (people/sq kilometer)	LandScan™ 2010 Global Population Dataset
Distance to settlement (km)	Forest inventory data in Changbaishan Mountain
Distance to road (including primary and secondary roads) (km)	Forest inventory data in Changbaishan Mountain
Detection covariates	
10‐day interval (categorical)	Cameras
30‐day interval (categorical)	Cameras
60‐day interval (categorical)	Cameras
10‐day interval (continual)	Cameras
30‐day interval (continual)	Cameras
60‐day interval (continual)	Cameras
Trail width	Field survey
Quadratic of trail width	Field survey
Camera effort	Camera days

These covariates were calculated in 5 scales, including radius of 0.5 km, 1 km, 1.5 km, 2 km, and 3 km area around the camera location

#### 2.2.2 Ungulate relative abundance model

Once we selected the best detection model for each season and each species, we then estimated the relative abundance for the three ungulate species with environmental and anthropogenic covariates based on previous studies of large carnivores and tigers (Carter et al., 2012; Hebblewhite et al., 2014). These environmental covariates were classified into two broad categories including abiotic covariates (elevation [m], slope [degrees], northness [cos(aspect)], distance to frontier [km], distance to river [km]), biotic covariates (forest type), and anthropogenic covariates (human activity, cattle activity, road density, human population density, distance to settlement [km], distance to road [km]) (Table 1). We used distance to Sino‐Russia frontier as a covariate to represent the potential for a positive effect of proximity to Russia on prey relative abundance, given the higher wild prey abundance in Russia, and recovering dynamics of tigers. We provide detailed explanations of all covariates in Table 1. As the scale of the effect of the environmental factors might differ between the different ungulate prey species (Harmsen, Foster, Silver, Ostro, & Doncaster, 2011), we created buffers with a radius of 0.5, 1, 1.5, 2, and 3 km around each camera site for elevation, slope, aspect, road density, human population density, and the forest type extraction similar to previous studies in the region on Amur tigers (Hebblewhite et al., 2014). As with the detection covariates, we conducted Pearson correlation test among covariates to avoid collinearity (Zuur, Ieno, & Elphick, 2010). We then selected the best scale for each of these 6 covariates with the lowest AIC of corresponding model.

After identifying the best fitting, scale‐specific environmental covariates, we established the best global model (Doherty et al., 2012) with all selected covariates for abundance holding detection probability as the full model identified above. Kéry and Royle (2015) noted the challenges in selecting the appropriate count distribution for N‐mixture models among three alternative distributions (Poisson, negative binomial, and zero‐inflated Poisson) for abundance due to the common “good fit/bad prediction dilemma” in analysis. We therefore fit the global N‐mixture model with the three distributions (Joseph et al., 2009). To determine the appropriate count model, we compared these three distributions using AIC for predictive ability and evaluated model fit using a goodness‐of‐fit test for the full model by bootstrapping 1,000 times (Kéry & Royle, 2015). If none of them passed the goodness of fit derived from unstructured overdispersion (large variance rather than structural deficiency in the mean structure of the model), we selected the most appropriate count distribution based on residual diagnostics and maps of predicted versus observed fit (Kéry & Royle, 2015). After selecting the best fitting count model type, we fit all combinations of covariates for abundance subsets and keep detection subset consistent as the best subset to establish the candidate models for model selection. Finally, we selected the top model based on AIC (Burnham & Anderson, 2002) guarding against including uninformative parameters following Arnold (2010). We did not consider model averaging when parameters within ≤2 delta AIC units were not statistically significant (Arnold, 2010).

### 2.3 Modeling tiger occupancy

We next used occupancy models to assess the relative effect of prey abundance (from the N‐mixture models developed above) and human disturbance covariates on the spatial variability in tiger occurrence during summer and winter seasons. Given the home range size of tigers in our study area and that tigers might move between China and Russia and hence would not always be exposed to the camera trap site, “occupancy” refers to relative use by tigers (Steenweg et al., 2018). We developed occupancy models again based on the 3.6 km^2^ grid and divided data to multioccasions within each season by defining 30 days as one occasion and established the encounter history at each site *i* using 1 for detected and 0 for undetected. All occupancy analyses were conducted in the R package “unmarked” (Fiske & Chandler, 2011) with standardized quantitative covariates.

#### 2.3.1 Tiger detection probability

Following the same rationale as for N‐mixture models, we first developed the best fitting detection probability model selection without occupancy covariates. The detection covariates included site covariates (average camera days), the number of cameras for each 3.6 × 3.6 km grid, trail width and the quadratic of trail width, and the time period as the observation covariate. Similar to the ungulate models, we took time period as a categorical covariate and a continuous covariate both with 30‐ or 60‐day intervals for univariate models. We then screened detection covariates for collinearity and then used AIC to select the appropriate time period covariate for modeling the detection process. We then conducted model selection based on AIC to find the top model for detection probability.

#### 2.3.2 Tiger occupancy model

To test how ungulate species, human, and cattle activities influenced tiger occurrence, we used the relative abundance predicted by the three ungulate species N‐mixture model above as covariates. For humans and cattle, we measured their activity frequency by the number of humans and cattle detections recorded by our cameras. We did not include forest cover type and nonbiotic covariates as these effects were accounted for already in N‐mixture models, and here we focused on testing biotic effects of wild prey, domestic preys, and humans on tiger occupancy. Similarly, to above, we screened potential covariates for collinearity and used AIC to select the top tiger occupancy model. We examined the model fit by goodness‐of‐fit test with 1,000 bootstrapping for comparing the observed data and expected data under the model (Kéry & Royle, 2015).

### 2.4 Occupancy–abundance relationship

The occurrence of tigers should positively relate to abundance through theoretical occurrence–abundance relationships (Boyce et al., 2016). Therefore, the factors affecting occurrence should also influence tiger abundance as well. We used quantile regression (Cade & Noon, 2003) to test for a positive relationship between the predicted occupancy and predicted density (Boyce et al., 2016). We used quantile regression because occupancy may only be expected to positively correlate to density at high levels of occupancy, that is, a triangular wedge‐shaped relationship between occupancy and abundance may be expected (Boyce et al., 2016). Density data were obtained from previous SCR modeling at each camera stations in the same area (Xiao et al., 2016), and the occupancy probability data were obtained from our occupancy prediction above at each camera stations. All analysis conducted in R package “quantreg” (Koenker, 2016).

### 2.5 Tiger–ungulate–human temporal (daily) overlap

To explore the temporal interactions of tigers with prey and humans, we used the time of detection from camera data to estimate the probability of occurrence on a daily temporal cycle for tigers, three prey species, humans, and cattle for each season. The probability density of species activity pattern was calculated based on the kernel density estimate and used to measure the daily overlap index Δ between two species, which range from 0 (no overlap) to 1 (complete overlap). We applied the estimator Δ4 due to our sample sizes larger than 100 and estimated the confidence intervals by 10,000 bootstrap samples using R package “overlap” (Linkie & Ridout, 2011; Ridout & Linkie, 2009).

## 3 RESULTS

We amassed a total of 53,347 camera days (24,771 in winter and 28,576 in summer). The average days per camera station were 152 in winter and 172 in summer. We obtained 276 independent events of tiger (162 in summer, 114 in winter), 707 of wild boar (574 in summer, 133 in winter), 871 of roe deer (641 in summer, 230 in winter), 1,378 of sika deer (992 in summer, 386 in winter), 11,638 of human (9,052 in summer, 2,586 in winter), and 1,428 of cattle.

### 3.1 Prey relative abundance

#### 3.1.1 Sika deer

The best fitting detection probability model for sika deer in both seasons varied among 10‐day time periods and was strongly influenced by sampling effort (camera days, for summer *β* = 0.16, *SE* = 0.06, for winter *β* = 0.27, *SE* = 0.13) and by a quadratic effect of trail width such that detections increased on wider roads up to a plateau of about 4 m (Table 2; Appendix S1: Table S2A). For both seasons, models within <2 delta AIC units contained additional, uninformative (i.e., nonsignificant) parameters and so we only report here the best‐fit top‐ranked model (Appendix S1: Table S1). Though Poisson, negative binomial, and zero‐inflated Poisson models all lacked goodness of fit in both seasons (*p* < 0.05), we did not find strong spatial patterns in residuals of the Poisson and the zero‐inflated Poisson models. Therefore, we determined lack of goodness of fit was caused by unstructured overdispersion (Kéry & Royle, 2015). Based on the residual diagnostics and maps (W. Xiao, unpublished data), we used the more parsimonious Poisson abundance models.

**Table 2:** The top N‐mixture models for the three prey species of Amur tigers in Hunchun Nature Reserve, China, 2012–2014, showing the covariates for detection (*p*) and relative abundance (*λ*) subset in summer and winter seasons. The best scales (the radius) for scale‐dependent covariates were displayed in parentheses

Species	Seasons	Parameters	Covariates in top models
Sika deer	Summer	*p*	10‐day interval (categorical), camera days, the quadratic of trail width
		*λ*	Distance to frontier, elevation (3 km), slope (3 km), human density (2 km), northness (3 km), distance to road, cattle, forest type (3 km)
	Winter	*p*	10‐day interval (categorical), camera days, the quadratic of trail width
		*λ*	Elevation (3 km), slope (3 km), human density (3 km), distance to road
Wild boar	Summer	*p*	10‐day interval (categorical), camera days
		*λ*	Distance to frontier, elevation (0.5 km), slope (3 km), northness (0.5 km), human activity, distance to river
	Winter	*p*	10‐day interval (categorical), camera days
		*λ*	distance to frontier, northness (2 km), human activity, distance to river, forest type (0.5 km)
Roe deer	Summer	*p*	10‐day interval (categorical), trail width
		*λ*	distance to frontier, elevation (1 km), human density (0.5 km), northness (3 km), human activity, cattle, distance to river, forest type (1 km)
	Winter	*p*	10‐day interval (categorical), trail width
		*λ*	Distance to frontier, road density (1.5 km), forest type (1 km)

**Figure 3:**
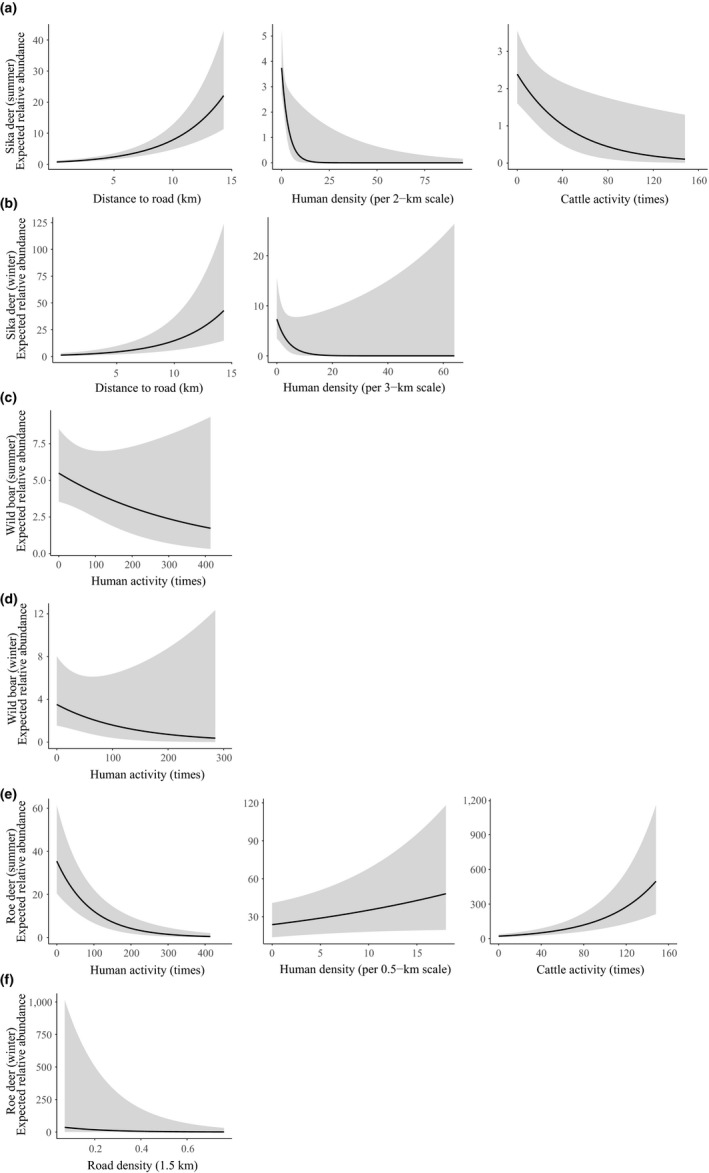
Relative abundance of Amur tiger prey including (a) sika deer in summer season, (b) sika deer in winter season, (c) wild boar in summer season, (d) wild boar in winter season, (e) roe deer in summer season, (f) roe deer in winter season with 95% confidence intervals (gray zones) in Hunchun Nature Reserve, China, 2012–2014

**Figure 4:**
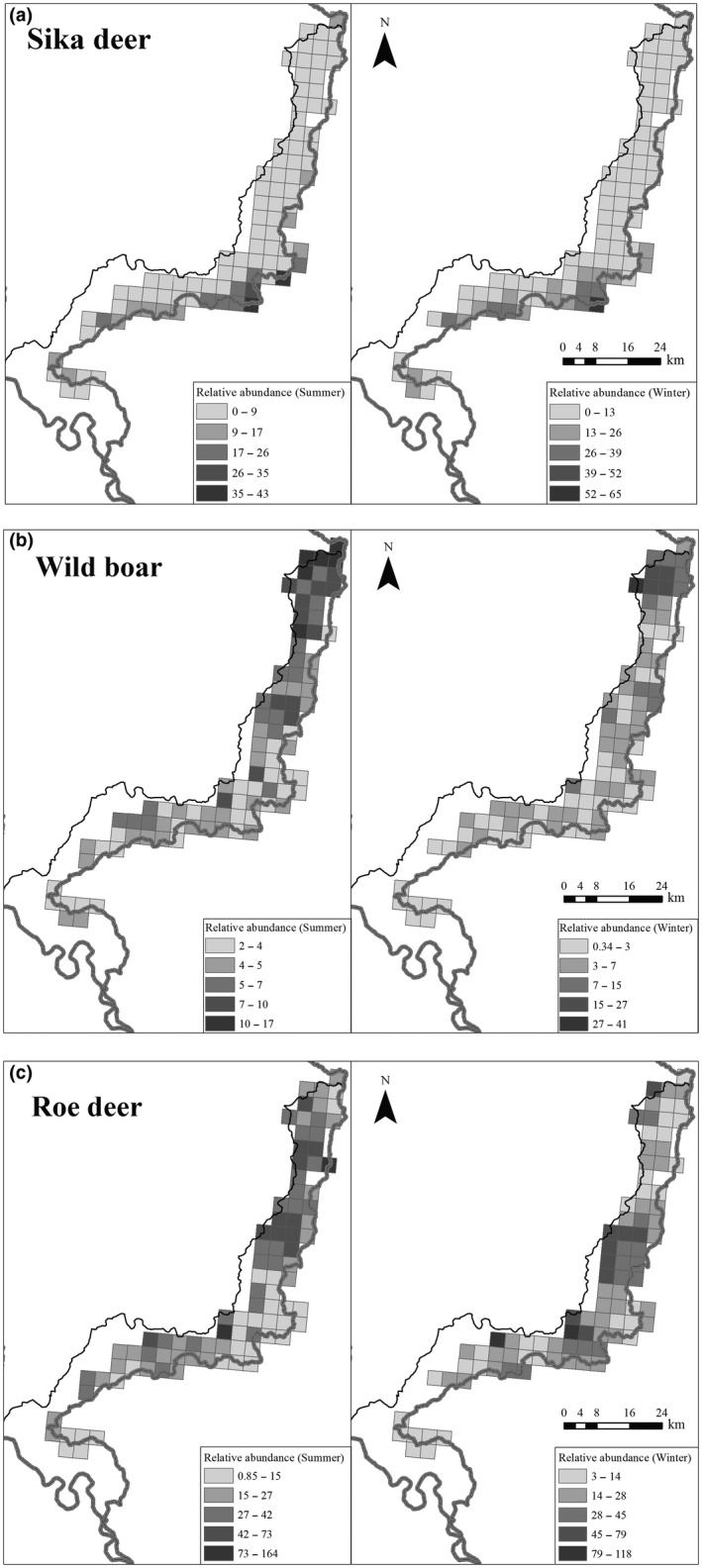
Estimates of spatial relative abundance in summer (left) and winter (right) in Hunchun Nature Reserve, China, 2012–2014 for Amur tiger prey: (a) sika deer; (b) wild boar; (c) roe deer. The prediction is based on the average relative abundance value in each tiger occupancy grid

In summer, the top model for sika deer abundance was a function of distance to frontier, elevation (3‐km scale), slope (3‐km scale), human density (2‐km scale), northness (3‐km scale), distance to road, cattle, and forest (3‐km scale) (Table 2; Appendix S1: Tables S1A and S2A; Figure 4a). Sika deer were more abundant in southern aspects (*β* = −0.21, *SE* = 0.06) of mixed Korean pine‐deciduous forest (*β* = 2.01, *SE* = 0.21, see Appendix S1: Figure S1B) with lower elevation (*β* = −0.84, *SE* = 0.10) but steep slope (*β* = 0.40, *SE* = 0.07) (Appendix S1: Table S2A). Meanwhile, sika deer relative abundance was higher further from roads (*β* = 0.69, *SE* = 0.06) and closer to the Sino‐Russian frontier (*β* = −0.29, *SE* = 0.07, see Appendix S1: Figure S1A) with lower human density (*β *= −2.56, *SE* = 0.86) and cattle occurrence (*β* = −0.37, *SE* = 0.11) (Figure 3).

During winter, elevation (3‐km scale), slope (3‐km scale), human density (3‐km scale), distance to road were the covariates in the top model for sika deer abundance (Table 2; Appendix S1: Tables S1A and S2A; Figure 4a). Similar to summer, sika deer were more abundant in the area far from roads (*β* = 0.71, *SE* = 0.07) with steep slopes (*β* = 0.61, *SE* = 0.07), lower elevations (*β* = −0.74, *SE* = 0.10), and human densities (*β* = −1.71, *SE* = 0.71) in winter (Figure 3; Appendix S1: Tables S1A and S2A).

#### 3.1.2 Wild boar

For both seasons, the best wild boar detection models varied among 10‐day time periods and were correlated with camera days (*β* = 0.09, *SE* = 0.06 in summer, *β* = −0.11, *SE* = 0.09 in winter, see Table 2; Appendix S1: Table S2B). Again, models within <2 delta AIC only differed mostly in addition of uninformative parameters (Appendix S1: Table S1A), so we report here only the top model. Poisson, negative binomial, and zero‐inflated Poisson models all showed lack of fit (*p* < 0.05) due to overdispersion indicating by our posterior predictive checks. Similar to the sika deer model, we chose the Poisson model based on comparing the three models’ residual diagnostics and maps (W. Xiao, unpublished data).

In summer, the top model of wild boar abundance was a function of distance to frontier, human activity, elevation (0.50‐km scale), slope (3‐km scale), northness (0.50‐km scale), and distance to river (Table 2; Appendix S1: Tables S1A and S2B; Figure 4b). The relative abundances of wild boar increased with the distance to Sino‐Russian frontier (*β* = 0.11, *SE* = 0.05) and fewer human activities in the forest (*β* = −0.18, *SE* = 0.08) (Figure 3; Appendix S1: Table S2B, Figure S1A). Wild boar was more abundant in southerly aspects (*β* = −0.14, *SE* = 0.07) and far from rivers (*β* = 0.13, *SE* = 0.05).

In winter, the distance to frontier, northness (2‐km scale), human activity, distance to river, and forest type (0.5‐km scale) (Table 2; Appendix S1: Tables S1A and S2B; Figure 4b) best predicted boar abundance. The relative abundances of wild boar increased with the distance to Sino‐Russian frontier (*β* = 0.28, *SE* = 0.06) and fewer human activities in the forest (*β* = −0.29, *SE* = 0.17) (Figure 3; Appendix S1: Table S2B, Figure S1A). In contrast to summer, wild boar exhibited opposite relationships with aspect (*β* = 0.32, *SE* = 0.10) and distance to rivers (*β* = −0.19, *SE* = 0.09) in winter. Wild boar preferred the mixed Korean pine‐deciduous forest (*β* = 2.42, *SE* = 0.33) (Appendix S1: Figure S1B).

#### 3.1.3 Roe deer

In summer and winter seasons, the detection probabilities varied among 10‐day time periods and increased in narrower trails (in summer *β* = −0.02, *SE* = 0.04, in winter *β* = −0.16, *SE* = 0.08, see Table 2; Appendix S1: Table S2C). Poisson, negative binomial, and zero‐inflated Poisson models all failed in goodness‐of‐fit test due to overdispersion rather than structural failure in two seasons (*p* < 0.05). Again, we chose the Poisson abundance model based on the residual diagnostics and maps (W. Xiao, unpublished data).

During summer, distance to frontier, elevation (at a 1‐km scale), human density (at a 0.50‐km scale), northness (at a 1‐km scale), human activity, cattle activity, distance to river, forest type (at a 1‐km scale) were the covariates in the top models of roe deer abundance (Table 2; Appendix S1: Tables S1A and S2C; Figure 4c). In summer, their relative abundance increased with the distance to frontier (*β* = 0.22, *SE* = 0.04) and river (*β* = 0.12, *SE* = 0.03). Roe deer were more abundant in southerly aspects (*β* = −0.19, *SE* = 0.05), in broadleaf deciduous forest (*β* = 3.20, *SE* = 0.19, Appendix S1: Figure S1B), at higher elevations (*β* = 0.23, *SE* = 0.05), at higher human density (*β* = 0.07, *SE* = 0.03), and at higher cattle activity occurrence (*β* = 0.37, *SE* = 0.03), but declined in areas of high human activity (*β* = −0.69, *SE* = 0.09) (Figure 3; Appendix S1: Table S2C).

In winter, the covariates in the top model of roe deer abundance included distance to frontier, road density (1.5‐km scale), and forest type (1‐km scale) (Table 2; Appendix S1: Tables S1A and S2C; Figure 4c). Roe deer were more abundant in the mixed Korean pine‐spruce forest (*β* = 3.15, *SE* = 1.09) with lower road density (*β* = −0.78, *SE* = 0.10) and at greater distances from frontier (*β* = 0.75, *SE* = 0.08) (Figure 3; Appendix S1: Table S2C, Figure S1A). Besides road density, no other human covariates affected roe deer relative abundance in winter.

### 3.2 Tiger occupancy

**Figure 5:**
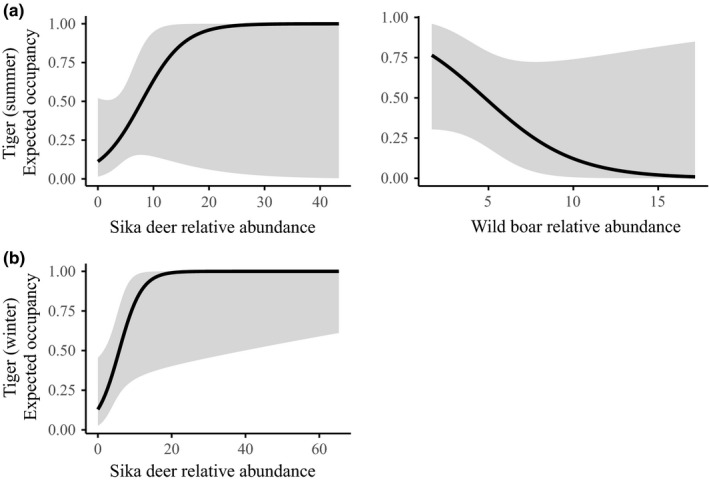
Amur tiger occupancy probabilities with 95% confidence intervals (gray zones) in Hunchun Nature Reserve, China, 2012–2014 in top models in (a) summer and (b) winter seasons as a function of covariates

**Figure 6:**
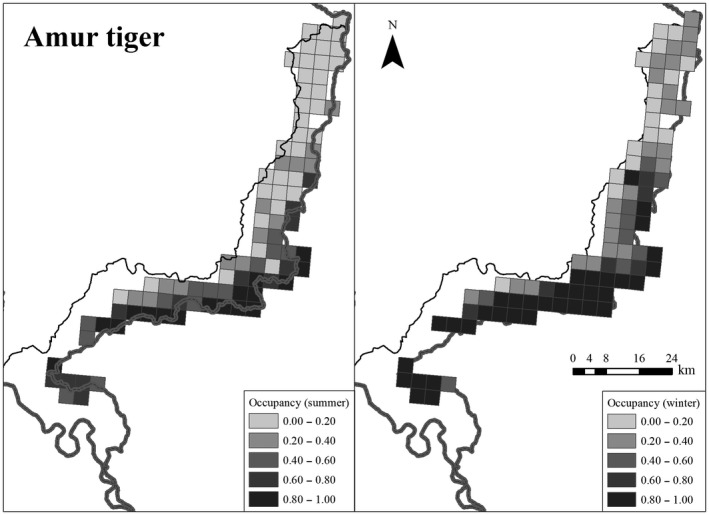
Spatial occupancy predictions for Amur tiger in summer (left) and winter (right) in Hunchun Nature Reserve, China, 2012–2014

In summer, the detection probabilities were correlated with the number of camera days (*β* = 0.39, *SE* = 0.17), a quadratic effect of trail (3.6 m trail width was the maximum), and varied among 60‐day time periods (Appendix S1: Tables S1B and S2D). Tiger occupancy probability increased with increasing relative abundance of sika deer (*β* = 2.07, *SE* = 1.07) but decreased with increasing wild boar abundance (*β* = −1.14, *SE* = 0.54) (Figures 5 and 6; Appendix S1: Table S2D). The goodness‐of‐fit test (*p* = 0.19) indicated model adequacy. In winter, the detection probabilities were positively correlated with the camera days (*β* = 0.44, *SE* = 0.34), the number of cameras (*β* = 0.46, *SE* = 0.17), and trail width (*β* = 0.60, *SE* = 0.18) and varied among 30‐day time periods (Appendix S1: Table S2D). During winter, the probability of tiger occupancy increased with sika deer's relative abundance (*β* = 3.52, *SE* = 1.70) (Figures 5 and 6; Appendix S1: Table S2D). There were no direct effects of humans in any of the top seasonal tiger occupancy models (Appendix S1: Table S2D). The *p*‐value = 0.38 for the goodness‐of‐fit test for the winter occupancy model confirmed model adequacy. Models within <2 delta AIC only differed mostly in addition of uninformative parameters in both seasons.

### 3.3 Tiger occupancy–abundance relationship

**Figure 7:**
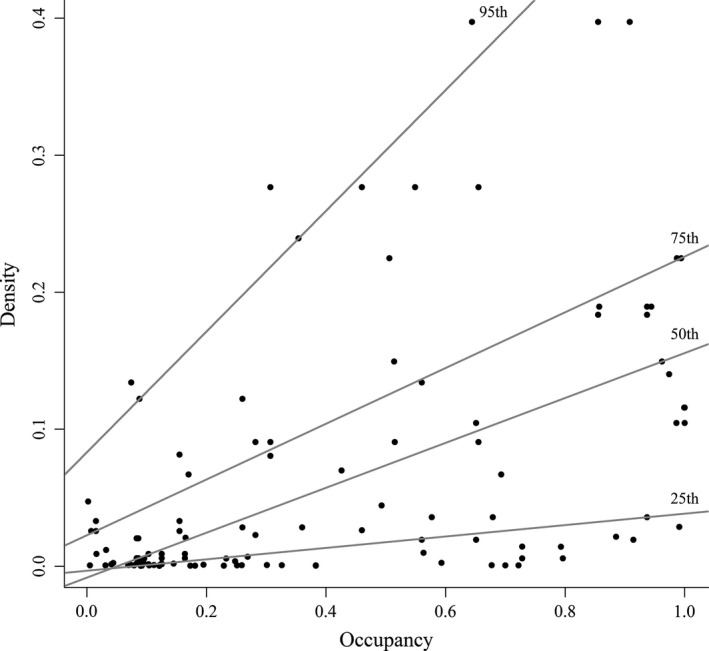
The estimated density (individuals/25 km^2^) of Amur tiger in Hunchun Nature Reserve, China, based on spatial explicit capture–recapture model illustrated a triangular relationship with predicted occupancy probability, showing 25th, 50th, 75th, 95th percentiles of density according to the quantile regression of density and occupancy

The occupancy probability of Amur tiger illustrated a significant positive correlation with density, with the correlation becoming stronger at higher percentiles of tiger density in the expected triangular, wedge‐shaped pattern (Figure 7). At the 95th quantile (upper limit) of tiger density, the coefficient with occupancy was the strongest (*β* = 0.44, CI = 0.29–0.75), compared with the 75th quantile (*β* = 0.20, CI = 0.19–0.58, *p* < 0.05), the 50th quantile (*β* = 0.16, CI = 0.15–0.22, *p* < 0.05), and the 25th quantile (*β* = 0.04, CI = 0.02–0.12, *p* < 0.05) (Figure 7).

### 3.4 Tiger–ungulate–human temporal (daily) overlap

**Figure 8:**
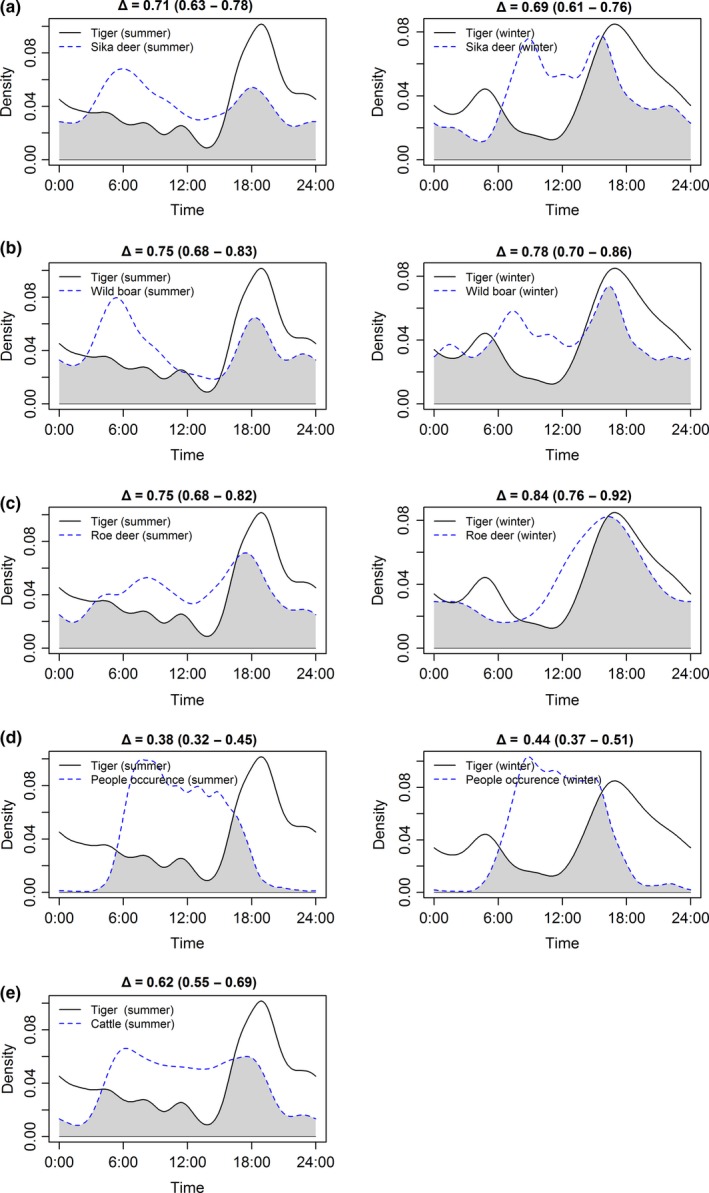
Daily activity patterns and overlaps between Amur tigers and (a) sika deer; (b) wild boar; (c) roe deer; (d) human activity; (e) cattle in Hunchun Nature Reserve, China, 2012–2014 in summer and winter seasons. The *x*‐axis presents daily 24 hr, and the *y*‐axis is the kernel density estimation. The black line and the gray dash line present kernel density estimation of daily activity pattern for tigers and corresponding species or human disturbance, respectively. Δ is the overlap index between tigers and corresponding prey species or human with 95% confidence interval showing inside the parentheses. The gray area corresponding to the coefficient of overlap

Overall, tigers showed higher overlap with ungulate prey, especially with roe deer (75% in summer and 84% in winter) and wild boar (75% in summer and 78% in winter). With respect to humans, tigers showed lower overlap with human activity than prey, and consistent with expectations about seasonal differences, lower overlap in summer (38%) than winter (44%), though the magnitude of the difference was not significantly different due to confidence interval overlap (Figure 8).

## 4 DISCUSSION

Humans appeared to primarily negatively affect tigers indirectly via human influences on their prey in a recolonizing population in HNR. Tigers avoided humans temporally during the day equivalently between summer and winter, but they did not spatially avoid human activity in the best occupancy model. Human‐related disturbance metrics strongly decreased the relative abundance of all three of the primary ungulate prey of tigers. These three prey species correlated strongly with tiger's spatial and temporal activity in both seasons. Thus, similar to other large carnivores (Carter et al., 2012; Riley et al., 2003; Smith, 2002), both humans and prey demonstrated important effects on tiger occurrence both spatially and temporally. Therefore, for recovering tiger population in Northeast China, more conservation actions should focus on the influence of humans on tigers through their prey species.

As the response of three prey species for human‐related disturbance varied in different scales and levels, humans had an important impact on tigers mediated through their indirect effects on prey in HNR. The relative abundance of the three ungulate species in our study area was shaped by a trade‐off between food availability and human disturbance (e.g., predation risk) (Hebblewhite & Merrill, 2009). Food abundance for ungulates is generally greater at lower elevations, in broadleaf forest cover types, and on southerly aspects, especially during winter (Hebblewhite, Merrill, & McDermid, 2008; Yokoyama, Kaji, & Suzuki, 2000), and these covariates were positively related to relative abundance of ungulates in our study. All three ungulate showed preferences for forest types that likely relate to their food habits, echoing similar results for these same ungulate species across the border in nearby Russia (Hebblewhite et al., 2014).

There was ample evidence that human activity mostly had negative effects on relative abundance of ungulates, consistent with considering humans as important predators of ungulates in this system (Li et al., 2016; Zhang, Zhang, & Stott, 2013). For example, transboundary efforts have removed more than 10,000 snares in HNR between 2001 and 2007, which were placed primarily to snare ungulates and other wildlife. Hound hunting for ungulates is also common. As expected under such hunting pressure, distance to road was positively related to sika deer relative abundance in both seasons (Figure 3). Roads may act as movement barriers, and increase vehicle collisions but probably have the biggest effect on prey through accessibility of humans to the forest for poaching (Proffitt et al., 2013). Sika deer might select areas far from roads in summer when resources are more readily available, while limited food resources in winter constrain sika deer distribution (Sakuragi et al., 2003). In winter, road density within a radius of 1.5 km decreased roe deer relative abundance, again consistent with poaching access that usually happened in winter season. Our results are consistent with Li et al (2016) study showing that the negative effects of road on tiger's prey. In addition, wild boar and roe deer negatively responded in similar ways as sika deer to human activity, which is possible that frequent human activity in the forest disturbed prey and increased mortality through snaring and hound hunting (Soh et al., 2014). One curious result we found was higher sika deer relative abundance close to the Sino‐Russian frontier, while wild boar relative abundance increased with distance to frontier. There are two main reasons for these patterns. First, previous studies report higher densities of Sika deer in Russia, where they have been expanding in recent decades, which probably fits with the higher sika deer abundance along the frontier in our results. Secondly, our results for wild boar likely reflect their preference for cropland that was usually far from frontier, more on the western side of our study area.

There was also temporal partitioning among prey species in our study that was consistent with indirect effects of human activity. Cattle had significantly higher daily activity overlap with sika deer and roe deer than wild boar. Cattle activity was also negatively correlated with the relative abundance of sika deer, potentially due to resource competition (Ripple et al., 2015). In contrast, higher cattle and human densities seemed to encourage roe deer abundance (Figure 3), and had no consistent effect on wild boar. In our study area, there may also be competition between roe deer and sika deer leading to ecological niche differentiation (Aramilev, 2009). Roe deer thrive in human‐dominated landscapes probably due to high quality forage and cover in field‐forest edges (Jiang, Ma, Zhang, & Stott, 2010; Torres, Carvalho, Panzacchi, Linnell, & Fonseca, 2011). Alternatively, roe deer and wild boar might potentially benefit from a human shield effect (Berger, 2007; Rogala et al., 2011). Regardless, effects of cattle on wild ungulates were stronger in summer, as livestock generally were removed from HNR during winter.

Tiger occupancy was correlated with the relative abundance of all three prey species both spatially, and temporally. Tiger spatial occupancy was positively correlated to sika deer abundance in both seasons (Figure 5; Appendix S1: Table S2D) supporting previous studies of habitat selection of tigers (Hebblewhite et al., 2014; Wang et al., 2016, 2018 ; Xiao et al., 2014) as well as diet studies that confirmed the importance of sika deer to the diet of tigers year‐round (Kerley et al., 2015). In contrast to sika deer, wild boar negatively affected tiger spatial occupancy in summer, but had no effect during winter. Wild boar also showed stronger temporal overlap with tigers in winter than in summer (Figure 8). In southern Russia adjacent to our study area, Kerley et al. (2015) found the percent biomass contribution from wild boar in winter was much more than in summer. Thus, a seasonal diet change may explain the difference from our study and others regarding avoidance of wild boar during summer. Alternatively, wild boar were mainly found in the northwest region of our study area while tigers were mostly centered on southeast region leading to low spatial overlap (Figures 4b and 6). Thus, nonequilibrial recolonization dynamics may explain the apparent avoidance of areas of high wild boar densities, contrary to results of previous studies that consistently show wild boar as the main prey of tigers. As tiger populations continue to expand in our study area and China, as a whole, wild boar may prove to be a more important prey. Given the nonequilibrial recovery of tigers in our study area, it is likely that tigers and their prey do not yet occur in their most suitable habitats. Even when tigers fully recover in our study area, there will still be natural fluctuations of food resources (e.g., mast year), and tigers and their prey may shift their habitat use according to the availability of food resources. Hence the situation might differ in a few years and tigers could select/prefer other prey species and the occupancy pattern of tigers and its preys might change as well. We found no direct spatial effect of cattle on tigers, despite previous studies that show tiger do prey on cattle occasionally in HNR (Soh et al., 2014) and that cattle occupancy directly discouraged tiger occurrence (Li et al., 2016; Wang et al., 2018). The difference is probably because our study addresses the influence of cattle on prey relative abundance, and prey was a key factor in determining the spatial occurrence of tigers. Though cattle did not directly affect the spatial occurrence of tigers, increasing cattle reduced relative abundance of sika deer (Wang et al., 2016) and increased ungulate poaching pressure (Soh et al., 2014). Consequently, cattle activity may have indirectly affected tiger occurrence by depressing wild prey.

Overall, our study demonstrates the important effects of ungulate prey on the spatial and temporal distribution of tiger occupancy in China. Avoidance of humans by large carnivores through temporal separation has been widely reported in human‐dominated landscapes (Carter et al., 2012; Riley et al., 2003). We show that through the negative effects of anthropogenic activities on prey abundance, human disturbance indirectly influenced tigers in HNR. While we were limited in being able to test for direct effects of humans on tiger mortality itself (the most important way to test for direct effects), our results are consistent with previous studies demonstrating that humans may have as important effects on tigers through their effects on poaching tiger prey (Chapron et al., 2008). This does not mean reducing direct poaching of tigers is not important, but also emphasizes the crucial importance of improved conservation of tiger prey, and stronger management of human activities. Thus, reducing human disturbances through more effective law enforcement (Linkie et al., 2015; Steinmetz, Chutipong, Seuaturien, Chirngsaard, & Khaengkhetkarn, 2010) and community engagement in conservation (Steinmetz, Srirattanaporn, Mor‐Tip, & Seuaturien, 2014) to promote ungulate prey recovery is recommended to facilitate tiger restoration in the larger potential habitat in China. For example, resettling local people living in tiger core habitat, reducing cattle activity, and reducing road construction (Li et al., 2016) to enhance prey species such as sika deer should encourage tiger occurrence. While our study showed that tigers did not avoid human‐related activities directly in terms of the spatial occupancy, it did show that they had a strong temporal avoidance (Figure 8). Tigers are dispersing from Russia to China despite the higher human disturbance levels there (Wang et al., 2016; Xiao et al., 2016). Temporal avoidance of human activity may be survival strategies to facilitate this population dispersal from low‐disturbance habitat to high disturbance. One potential weakness of our temporal activity analysis could be sample size, especially of tigers, but we obtained ample sample sizes of prey species (Frey, Fisher, Burton, Volpe, & Rowcliffe, 2017).

Our quantile regression supports the interpretation that tiger occupancy was correlated to previously published estimates of tiger density (Xiao et al., 2016). However, the relationship was only strong at higher densities and that some factors (e.g., prey availability) may be important in driving when tigers first occupy a site. The scale of occupancy model also influences the relationship of occupancy–abundance relationship (Kéry & Royle, 2015). It is not currently possible to incorporate spatial covariates in SCR models both because of our small recovering tiger population (e.g., small sample size) and technical limitations of SCR models for rare species. Regardless, our study confirms that there was a general positive relationship between occupancy and the highest densities of Amur tigers, similar to previous studies on other large carnivores such as grizzly bears (Boyce et al., 2016) and jaguars (Tôrres et al., 2012). Thus, our ability to test for factors affecting occupancy should be related to similar effects on tiger densities.

Our study demonstrated humans can affect tigers via their impacts on ungulate prey. Humans, as another potential predator for ungulate, disturbance induced by humans can affect tigers by reducing their prey abundance, increasing human–tiger conflict (Ripple et al., 2014, 2015 ). Tigers have great reproductive potential if poaching is reduced, and reproductive potential is driven by access to high densities of large ungulate prey (Chapron et al., 2008). To achieve the global aim of saving endangered big cats, recovering prey populations and reducing human‐related disturbance will be one crucial strategy (Sanderson et al., 2006). Conservation should also focus on direct threats on tigers such as poaching (Goodrich et al., 2008). Meanwhile, conservation efforts need to target on human‐induced threat on prey by enhancing dynamic prey population monitor and identifying related anthropogenic influences (Duangchantrasiri et al., 2016; Kawanishi et al., 2013).

## CONFLICT OF INTEREST

None declared.

## AUTHOR CONTRIBUTIONS

W. X., T. W., M. H., P. M, and J. G. conceived the ideas and designed methodology; L. F., W. X., and B. Z. collected the data; W. X., M. H., and H. R. analyzed and interpreted the data; W. X., M. H., T. W., and H. R. led the writing of the manuscript. All authors contributed critically to the drafts and gave final approval for publication.

## DATA ACCESSIBILITY

The datasets involve spatial locations of an endangered species, the Amur tiger, restricting us from making them publicly available. Some parts of the data are available from the corresponding author on specific request.
