# Supplementary material for: Relationships between humans and ungulate prey shape Amur tiger occurrence in a core protected area along the Sino‐Russian border
Source: Ecol Evol. 2018 Oct 30;8(23):11677–93. doi: 10.1002/ece3.4620 (PMC6303753; doi:10.1002/ece3.4620)

**Relationships between humans and ungulate prey shape Amur tiger occurrence in a core protected area along the Sino-Russian border**

# Appendices

**Appendix Table S1** Summary of model selection results for (A) ungulate N-mixture models; (B)tiger occupancy models, showing the effect of covariates on relative abundance (lam), occupancy probability (psi) and detection probability (p) for the models with ΔAIC<2.

1. Ungulate N-mixture model selection results
   1. Sika deer
      1. Summer

| Model^†^ | nPars | AIC | delta AIC | AICwt |
| --- | --- | --- | --- | --- |
| lam(DistFro+elev3k+slope3k+PeopDen2k+North3k+Dist2Road+Cattle+forest3k) | 32 | 5525.67 | 0 | 0.99 |

DistFro = distance to frontier, elev3k = elevation (radius of 3km), slope3k = slope (radius of 3km), PeopDen2k = human density (radius of 2km), North3k = northness (radius of 3km), Dist2Road = distance to road, cattle = cattle activity, forest3k=forest type (radius of 3km).

^†^The covariates for detection probability subset are 10-days interval (categorical), camera days, the quadratic of trail width

- - 1. Winter

| Model^†^ | nPars | AIC | delta AIC | AICwt |
| --- | --- | --- | --- | --- |
| lam(elev3k+slope3k+PeopDen3k+North1k+Dist2Road) | 28 | 2554.82 | 0 | 0.24 |
| lam(DistFro+elev3k+slope3k+PeopDen3k+North1k+Dist2Road) | 29 | 2555.02 | 0.20 | 0.22 |
| lam(DistFro+elev3k+slope3k+PeopDen3k+Dist2Road) | 28 | 2555.06 | 0.24 | 0.21 |
| lam(elev3k+slope3k+PeopDen3k+Dist2Road) | 27 | 2555.51 | 0.70 | 0.17 |

DistFro = distance to frontier, elev3k = elevation (radius of 3km), slope3k = slope (radius of 3km), PeopDen3k = human density (radius of 3km), North1k = northness (radius of 1km), Dist2Road = distance to road, forest3k=forest type (radius of 3km),

^†^The covariates for detection probability subset are 10-days interval (categorical), camera days, the quadratic of trail width

- 1. Wild boar
     1. Summer

| Model^†^ | nPars | AIC | delta AIC | AICwt |
| --- | --- | --- | --- | --- |
| lam(DistFro+elev05k+slope3k+North05k+Hum+Cattle+DistRiver) | 28 | 3824.83 | 0 | 0.21 |
| lam(DistFro+elev05k+slope3k+North05k+Hum+DistRiver) | 27 | 3825.5 | 0.66 | 0.15 |
| lam(DistFro+elev05k+slope3k+North05k+Cattle+DistRiver) | 27 | 3826.59 | 1.75 | 0.09 |

DistFro = distance to frontier, elev05k = elevation (radius of 0.5km), slope3k = slope (radius of 3km), North05k = northness (radius of 0.5km), Hum = human activity, Cattle = cattle activity, DistRiver = distance to river, forest05k=forest type (radius of 0.5km).

^†^The covariates for detection probability subset are 10-days interval (categorical), camera days

- - 1. Winter

| Model^†^ | nPars | AIC | delta AIC | AICwt |
| --- | --- | --- | --- | --- |
| lam(DistFro+PeopDen05k+North2k+Hum+DistRiver+forest05k) | 28 | 1649.19 | 0 | 0.20 |
| lam(DistFro+elev3k+PeopDen05k+North2k+Hum+DistRiver+forest05k) | 29 | 1649.30 | 0.11 | 0.18 |
| lam(DistFro+elev3k+North2k+Hum+DistRiver+forest05k) | 28 | 1649.79 | 0.60 | 0.14 |
| lam(DistFro+North2k+Hum+DistRiver+forest05k) | 27 | 1649.82 | 0.63 | 0.14 |

DistFro = distance to frontier, elev3k = elevation (radius of 3km), PeopDen05k = human density (radius of 0.5km), North2k = northness (radius of 2km), Hum = human activity, DistRiver = distance to river, forest05k=forest type (radius of 0.5km).

^†^The covariates for detection probability subset are 10-days interval (categorical), camera days

- 1. Roe deer
     1. Summer

| Model^†^ | nPars | AIC | delta AIC | AICwt |
| --- | --- | --- | --- | --- |
| lam(DistFro+elev1k+slope1k+PeopDen05k+North3k+Hum+Cattle+DistRiver+forest1k) | 31 | 6162.95 | 0.00 | 0.38 |
| lam(DistFro+elev1k+PeopDen05k+North3k+Hum+Cattle+DistRiver+forest1k) | 30 | 6163.64 | 0.68 | 0.27 |
| lam(DistFro+elev1k+slope1k+PeopDen05k+North3k+Hum+Cattle+DistRiver+Dist2Road+forest1k) | 32 | 6164.73 | 1.78 | 0.16 |

DistFro = distance to frontier, elev1k = elevation (radius of 1km), slope1k = slope (radius of 1km), PeopDen05k = human density (radius of 0.5km), North3k = northness (radius of 3km), Cattle = cattle activity, Hum = human activity, DistRiver = distance to river, Dist2Road = distance to road, forest1k=forest type (radius of 1km).

^†^The covariates for detection probability subset are 10-days interval (categorical), trail width.

- - 1. Winter

| Model^†^ | nPars | AIC | delta AIC | AICwt |
| --- | --- | --- | --- | --- |
| lam(DistFro+RoadDen1.5k+forest1k) | 25 | 1410.79 | 0.00 | 0.51 |
| lam(DistFro+RoadDen1.5k+PeopDen1k+forest1k) | 26 | 1410.83 | 0.04 | 0.49 |

DistFro = distance to frontier, RoadDen1.5k = road density (radius of 1.5km), PeopDen1k = human density (radius of 1km), forest1k=forest type (radius of 1km).

^†^The covariates for detection probability subset are 10-days interval (categorical), trail width

1. Tiger occupancy model selection
   1. Summer

| Model^†^ | nPars | AIC | delta AIC | AICwt |
| --- | --- | --- | --- | --- |
| psi(WB+SD) | 10 | 364.22 | 0.00 | 0.31 |
| psi(Hum+WB+SD) | 11 | 366.02 | 1.81 | 0.13 |
| psi(Cattle+WB+SD) | 11 | 366.12 | 1.90 | 0.12 |
| psi(RD+WB+SD) | 11 | 366.20 | 1.99 | 0.12 |

WB = wild boar, SD = sika deer, RD = roe deer, Hum = human activity, Cattle = cattle activity.

^†^The covariates for detection probability subset are 60-days interval (categorical), camera days, the quadratic of trail width.

- 1. Winter

| Model^†^ | NPars | AIC | delta AIC | AICwt |
| --- | --- | --- | --- | --- |
| psi(SD) | 12 | 250.78 | 0.00 | 0.37 |
| psi(WB+SD) | 13 | 252.61 | 1.83 | 0.15 |
| psi(RD+SD) | 13 | 252.63 | 1.86 | 0.15 |
| psi(Hum+SD) | 13 | 252.68 | 1.90 | 0.14 |

WB = wild boar, SD = sika deer, RD = roe deer, Hum = human activity.

^†^The covariates for detection probability subset are 30-days interval (categorical), the number of cameras, camera days, trail width.

**Appendix Table S2** Standardized covariate coefficients with corresponding standard errors and P-values for the top models explaining ungulate prey (A) sika deer; (B) wild boar; (C) roe deer relative abundance and, (D) Amur tiger occupancy and detection probability in summer and winter seasons.

1. **Sika deer**

| Summer | | | | Winter | | | |
| --- | --- | --- | --- | --- | --- | --- | --- |
| Covariates | Coefficients | SE | P-value | Covariates | Coefficients | SE | P-value |
| **Relative abundance** | | | | | | | |
| - | - | - | - | (Intercept) | 1.36 | 0.31 | <0.01 |
| Elevation (3km radius) | -0.84 | 0.10 | <0.01 | Elevation (3km radius) | -0.74 | 0.10 | <0.01 |
| Slope  (3km radius) | 0.40 | 0.07 | <0.01 | Slope (3km radius) | 0.61 | 0.07 | <0.01 |
| Human density (2km radius) | -2.56 | 0.86 | <0.01 | Human density (3km radius) | -1.71 | 0.71 | 0.016 |
| Distance to road | 0.69 | 0.06 | <0.01 | Distance to road | 0.71 | 0.07 | <0.01 |
| Northness (3km radius) | -0.21 | 0.06 | <0.01 | - | - | - | - |
| Cattle | -0.37 | 0.11 | <0.01 | - | - | - | - |
| Distance to frontier | -0.29 | 0.07 | <0.01 | - | - | - | - |
| BroDecid (3km radius) | 0.77 | 0.15 | <0.01 | - | - | - | - |
| BroMixKorPine (3km radius) | 2.01 | 0.21 | <0.01 | - | - | - | - |
| KorPineSpru (3km radius) | 0.81 | 0.52 | 0.120 | - | - | - | - |
| **Detection^†^** | | | | | | | |
| Camera days | 0.16 | 0.06 | <0.01 | Camera days | 0.27 | 0.13 | 0.034 |
| Trail width | 0.03 | 0.05 | 0.577 | Trail width | 0.22 | 0.07 | <0.01 |
| I(trail width^2) | -0.02 | 0.03 | 0.433 | I(trail width^2) | -0.05 | 0.03 | 0.181 |

BroDecid= Broadleaf deciduous forest, BroMixKorPine = Mixed Korean pine-deciduous forest, KorPineSpru = Korean pine-spruce forest

1. **Wild boar**

| Summer | | | | | Winter | | | |
| --- | --- | --- | --- | --- | --- | --- | --- | --- |
| Covariates | Coefficients | | SE | P-value | Covariates | Coefficients | SE | P-value |
| **Relative abundance** | | | | | | | | |
| (Intercept) | 1.61 | | 0.13 | <0.01 | - | - | - | - |
| Distance to frontier | 0.11 | | 0.05 | 0.014 | Distance to frontier | 0.28 | 0.06 | <0.01 |
| Northness  (0.5km radius) | -0.14 | | 0.07 | 0.042 | Northness (2km radius) | 0.32 | 0.10 | <0.01 |
| Human activity | -0.18 | | 0.08 | 0.030 | Human activity | -0.29 | 0.17 | 0.091 |
| Distance to river | 0.13 | | 0.05 | <0.01 | Distance to river | -0.19 | 0.09 | 0.026 |
| Slope  (3km radius) | | -0.20 | 0.04 | <0.01 | - | - | - | - |
| Elevation (0.5km radius) | | 0.31 | 0.05 | <0.01 | - | - | - | - |
| - | | - | - | - | BroDecid (0.5km radius) | 1.16 | 0.31 | <0.01 |
| - | - | | - | - | BroMixKorPine  (0.5km radius) | 2.42 | 0.33 | <0.01 |
| - | - | | - | - | KorPineSpru  (0.5km radius) | 1.78 | 0.42 | <0.01 |
| **Detection^†^** | | | | | | | | |
| Camera days | 0.09 | | 0.06 | 0.136 | Camera days | -0.11 | 0.09 | 0.225 |

BroDecid= Broadleaf deciduous forest, BroMixKorPine = Mixed Korean pine-deciduous forest, KorPineSpru = Korean pine-spruce forest

1. **Roe deer**

| Summer | | | | Winter | | | |
| --- | --- | --- | --- | --- | --- | --- | --- |
| Covariates | Coefficients | SE | P-value | Covariates | Coefficients | SE | P-value |
| **Relative abundance** | | | | | | | |
| Distance to frontier | 0.22 | 0.04 | <0.01 | Distance to frontier | 0.75 | 0.08 | <0.01 |
| Elevation (1km radius) | 0.23 | 0.05 | <0.01 | - | - | - | - |
| Human density (0.5km radius) | 0.07 | 0.03 | <0.01 | - | - | - | - |
| Northness (1km radius) | -0.19 | 0.05 | <0.01 | - | - | - | - |
| Human activity | -0.69 | 0.09 | <0.01 | - | - | - | - |
| Cattle | 0.37 | 0.03 | <0.01 | - | - | - | - |
| Distance to river | 0.12 | 0.03 | <0.01 | - | - | - | - |
| - | - | - | - | Road density (1.5km radius) | -0.78 | 0.10 | <0.01 |
| BroDecid (1km radius) | 3.20 | 0.19 | <0.01 | BroDecid (1km radius) | 2.98 | 1.06 | <0.01 |
| BroMixKorPine (1km radius) | 2.67 | 0.22 | <0.01 | BroMixKorPine (1km radius) | 2.00 | 1.07 | 0.062 |
| KorPineSpru (1km radius) | 1.83 | 0.30 | <0.01 | KorPineSpru (1km radius) | 3.15 | 1.09 | <0.01 |
| **Detection^†^** | | | | | | | |
| Trail width | -0.02 | 0.04 | 0.606 | Trail width | -0.16 | 0.08 | 0.039 |

BroDecid= Broadleaf deciduous forest, BroMixKorPine = Mixed Korean pine-deciduous forest, KorPineSpru = Korean pine-spruce forest

1. **Amur tiger**

| Summer | | | | Winter | | | |
| --- | --- | --- | --- | --- | --- | --- | --- |
| Covariates | Coefficients | SE | P-value | Covariates | Coefficients | SE | P-value |
| **Occupancy** | | | | | | | |
| (Intercept) | -0.27 | 0.52 | 0.597 | (Intercept) | 1.29 | 1.05 | 0.222 |
| Sika deer | 2.07 | 1.07 | 0.054 | Sika deer | 3.52 | 1.70 | 0.039 |
| Wild boar | -1.14 | 0.54 | 0.034 | - | - | - | - |
| **Detection** | | | | | | | |
| Camera days | 0.39 | 0.17 | 0.026 | Camera days | 0.44 | 0.34 | 0.202 |
| Trail width | 1.27 | 0.29 | <0.01 | Trail width | 0.60 | 0.18 | <0.01 |
| I(trail width^2) | -0.31 | 0.12 | <0.01 | - | - | - | - |
| - | - | - | - | No. Cameras | 0.46 | 0.17 | <0.01 |

**Appendix Fig. S1** Relative abundance of ungulate prey for Amur Tigers in Hunchun Nature Reserve, China, 2012 – 2014 used to predict in respect of (A) distance to frontier, (B) forest type under top models in summer and winter seasons. RD-S and RD-W present roe deer in summer and in winter respectively, WB-S and WB-W mean wild boar in summer and in winter respectively, and SD-S is sika deer in summer.
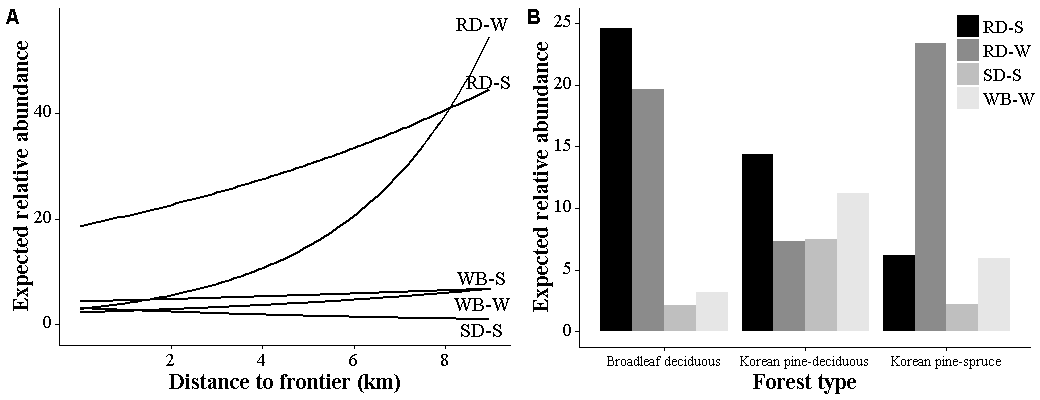

Supplement: Supplementary file 1 [file ECE3-8-11677-s001.docx]
